# Resources of emotional resilience and its mediating role in teachers’ well-being and intention to leave

**DOI:** 10.3389/fpsyg.2023.1305979

**Published:** 2023-11-21

**Authors:** Dalia Bagdžiūnienė, Aušra Kazlauskienė, Dalia Nasvytienė, Emilija Sakadolskis

**Affiliations:** ^1^Institute of Psychology, Vilnius University, Vilnius, Lithuania; ^2^Institute of Education, Siauliai Academy, Vilnius University, Šiauliai, Lithuania; ^3^Educational Research Institute, Vytautas Magnus University, Kaunas, Lithuania

**Keywords:** teacher, emotional resilience, job resources, self-efficacy, well-being, intention to leave

## Abstract

The continuing attention of scholars and practitioners to the teaching profession, teachers and teaching is based above all on the fact that societal progress is impossible without an effective education system. Teachers are the “soft” dynamic, and at the same time, a driving force in this constantly changing system, and research into the prerequisites for their effective performance requires constant attention. In this study, the main phenomenon under analysis is the emotional resilience of teachers–the internal capacity to adapt, manage or cope with emotionally demanding situations. The purpose was to investigate work-related and personal resources that contribute to teachers’ emotional resilience and its role in the links between resources, teacher well-being, and the intention to leave. Data were collected using convenience sampling and included 522 teachers working in Lithuanian primary and secondary schools. An online self-administered questionnaire consisted of scales that assessed teachers’ job resources, self-efficacy, well-being, and intention to leave. The research revealed that perceived workplace characteristics – performance feedback, autonomy, social support, and opportunities for professional growth–along with self-efficacy were positively related and predicted teacher emotional resilience. Emotional resilience was found to be a direct positive predictor of teacher well-being along with job resources and self-efficacy and have a mediating effect on the relationships between work-related resources and self-efficacy as independent variables and teacher wellbeing as a dependent variable. Contrary to well-being is teachers’ intention to leave a school, which is usually an undesirable outcome for an organization. The study revealed that this intention is negatively affected by job resources and self-efficacy, however emotional resilience did not impact teachers’ intention to quit. Based on the results, the article outlines avenues for further research and provides implications for strengthening teachers’ emotional resilience.

## 1 Introduction

Numerous studies report that teaching is emotionally demanding, and that emotional resilience was particularly important during the COVID-19 pandemic, when education systems were confronted with the challenges of distance learning and other pressures necessitating the search for optimum solutions. After the pandemic, the world did not return to the *status quo ante*. The world of work has changed rapidly and significantly, and constant change has become characteristic of the work of teachers. As with many other professions, teachers have had to adapt to new demands and conditions while striving for high quality teaching. There are also the everyday challenges of pupil behavior, learning difficulties, organizational concerns, or stressful communication situations (Cordingley and Crisp, 2020). In Lithuania recent developments include curriculum renewal in 2023, changes in the composition of the student body, and the full inclusion of pupils with disabilities or linguistic multiplicity to be implemented in 2024. This situation has not only affected teachers’ well-being, but also led to a significant number of teachers leaving their jobs, as in many other countries (See et al., 2020; Alves et al., 2021). Limited research reveals that teachers’ emotional resilience is one of the factors impacting their well-being and intention to leave, so it is appropriate to examine work and personal factors that enhance emotional resilience, as well as the implications for positive organizational and personal outcomes.

Positive emotions and the ability to maintain emotional balance help people cope with situations of extreme stress (Diener et al., 2020) and effectively manage day-to-day routine teaching processes when deciding what and how to teach (Sheppard and Levy, 2019). Emotional resilience contributes to positive teacher-student relationships (Hagenauer et al., 2015), cooperative classroom environments (Wang et al., 2020), positively impacts teachers’ emotional well-being (Näring et al., 2012), and professional lives in general (Chen, 2020). At the organizational level positive emotions can be significant in maintaining a stable staff, enhancing teachers’ commitment to the organization, and reducing instances of teachers intending to leave school (Lee et al., 2021). Emotional resilience can therefore be considered a psychological factor that can strengthen teachers’ well-being and their relationship with the organization. Another aspect of emotional resilience and its link to well-being and intention to leave is its role as a mediating psychological factor in the relationship between job and personal resources with well-being and intention to leave. To our knowledge, the role of emotional resilience as a mediator in the context of the problem under investigation has not been explored in detail.

We investigated the issue of teacher emotional resilience from several perspectives: firstly, we investigated work related and personal resources as antecedents of teacher emotional resilience, secondly, we examined the relationship between emotional resilience and teacher well-being and intention to leave, and, thirdly, we examined the role of emotional resilience as a mediating variable in the relationship between job and personal resources with teacher well-being, and intention to leave.

## 2 Theoretical framework and literature review

### 2.1 Teacher emotional resilience

According to Luthans (2002), resilience is a basic phenomenon in positive psychology and in employee behavior studies. In general resilience could be defined as “the positive psychological capacity to rebound, to ‘bounce back’ from adversity, uncertainty, conflict, failure or even positive change, progress and increased responsibility” (p. 702). Meta-analyses of resilience research (Britt et al., 2016; Hartmann et al., 2020) provide a wide variety of definitions of resilience, which tend to emphasize three aspects of this phenomenon: as mentioned, the first reflects the capacity of the individual to “bounce back” from a stressful situation (Luthans, 2002), the second is associated with the ability to utilize resources, to adapt to changes and to demonstrate positive behavioral transformation when resolving challenging situations (Luthar et al., 2000), and the third aspect highlights dynamic rather than static personal strength, which can be nurtured and developed (Baker et al., 2021).

In recent years research on teacher resilience has gained increased attention because, as Day (2017) states, “teaching is emotional work and that moral purpose, efficacy and agency are key parts of teachers’ positive professional identities, important to their lives, well-being and effectiveness” (p. 44). The phenomenon of teacher resilience has been analyzed extensively (Beltman, 2021), but research on their emotional resilience–the capacity to regain and maintain emotional equilibrium and a positive attitude in difficult emotional situations– has not been fully examined (Day and Hong, 2016).

Employees with high emotional resilience quickly regain their emotional balance in the face of stressful and complex situations at work and in their personal lives, and redirect their cognitive, emotional, and physical energies to cope with difficulties and adapt to change (Lloyd et al., 2016). Murden et al. (2018) define emotional resilience as the ability to successfully adapt to disruptions, to smooth out occupational stress and to “switch” from a state of resistance and coping to a state of growth and development. Grant and Kinman (2014) refer to this phenomenon as the ability to motivate oneself, to control impulses and regulate one’s mood.

According to a four-dimensional framework developed by Mansfield et al. (2012) emotional resilience is one of four teacher resilience types along with professional, motivational, and social resilience. This framework is based on the authors’ research with graduating pre-service and early career teachers. The emotional dimension of teacher resilience is defined as “emotional responses to teaching experiences, emotional management and coping with stress” (Mansfield et al., 2012, p. 362). It refers to the emotional responses to daily teaching experiences, emotion management and coping with stress; it includes the personal attributes, characteristics and/or strategies teachers employ in front of adversities, such as the ability to manage their emotions, to maintain emotional stability, not take things personally, having a sense of humor (Mansfield et al., 2012), being emotionally intelligent (Chan et al., 2008), enjoying teaching and having a feeling of personal fulfillment (Howard and Johnson, 2004; Mansfield et al., 2012).

It is appropriate to look at this phenomenon from two perspectives. The first relates to individual and organizational outcomes which emotional resilience impacts. Studies of employees in various occupations confirm that emotional resilience can enhance positive outcomes for employees and organizations, including well-being, job satisfaction, work engagement, performance, and retention (Grant and Kinman, 2014; Hartmann et al., 2020). But what is the importance of emotional resilience specifically for teachers, their well-being and their turnover intention?

Another perspective relates to emotional resilience resources. According to Fletcher and Sarkar (2013) it is important to analyze a person’s immediate environment, and “to develop the protective and promotive factors that individuals can proactively utilize to build resilience” (p. 18). Gu and Day (2007, 2013) point out that teacher resilience is a dynamic phenomenon influenced by many personal and environmental factors. Hartmann et al. (2020) also confirm that resilience-promoting factors are important elements of the resilience process. It is therefore pertinent to explore the impact of the work environment and personal characteristics on teachers’ emotional resilience.

### 2.2 Job resources and teacher emotional resilience

The concept of job resources was introduced and elaborated by Bakker and his colleagues (Bakker and Demerouti, 2007; Bakker et al., 2010; Bakker and de Vries, 2021) and refers to the physical, psychological, social, or organizational aspects of the job that help employees achieve work goals and encourage personal growth and development (Hakanen et al., 2008; Collie et al., 2020; Granziera et al., 2021; Chen and Lee, 2022). The Job Demands-Resources Theory (Bakker and Demerouti, 2017) examines the interplay between job demands and available resources that workers can use to fulfill demands and achieve professional goals. Positive employee and organizational outcomes are possible if employees have sufficient job-related (performance feedback, autonomy, social support, opportunities for professional growth) and personal (adaptability, optimism, self-efficacy) resources. Adequate provision of both types of resources strengthens employee work motivation and engagement (Xanthopoulou et al., 2007; Christensen et al., 2020), and can also enhance work-related resilience (Boldrini et al., 2019; Chen and Lee, 2022). However, the implications of these factors specifically for teacher emotional resilience have not been sufficiently investigated (Day and Hong, 2016).

Xanthopoulou et al. (2007), building on Hobfoll’s (1989) Conservation of Resources Theory, were among the first to incorporate personal resources into the Job Demands-Resources model. Personal resources build a person’s motivational potential, enhance work engagement and help to achieve positive outcomes for the individual and organization. One of the personal resources is self-efficacy. The construct’s author Bandura (1977, 2012) defined self-efficacy as people’s beliefs in their capabilities to produce given attainments (2012), “a generative capability in which cognitive, social, emotional, and behavioral subskills must be organized and effectively orchestrated” (1997, p. 37) in various contexts. Pahwa and Khan (2022) state that “Self-efficacy means that a person has confidence in his abilities to perform a task in a particular situation” (p. 221) and describe it as a key personal antecedents of emotional resilience in adults along with purposefulness and meaning, self-awareness, problem solving, learning attitude and other personal factors.

Both self-efficacy and resilience are important for personal adaptation, and both are related yet autonomous constructs. The former is more rational, conditioned by past experiences, while the latter expresses emotional readiness to withstand present difficulties. The differences between these phenomena can be seen from two perspectives: the situations in which they occur and their role in regulating behavior. Tait (2008) emphasizes that the difference between resilience and self-efficacy is that resilient individuals are able to respond and manage stressful situations, whereas efficacious individuals are proactive in a variety of circumstances. Resilience refers to the inner capacity to adapt and maintain emotional equilibrium, while personal efficacy beliefs have strong motivational potential, encouraging people to show initiative, to engage in new activities, and to pursue more complex goals. As Schwarzer and Warner (2012), resilience is involved in regulating behavior when a person is exposed to stressors or adverse circumstances, while self-efficacy operates in a wide range of circumstances, even when a person is not experiencing challenges or trauma. Beliefs about personal efficacy, together with other intrinsic and extrinsic motivators of behavior help to answer the question of whether a person will engage in an activity, while emotional resilience determines how, with what emotional reactions a person will cope with challenging situations, and to what extent they will be able to maintain emotional equilibrium and move on to finding rational solutions.

Numerous studies have found that self-efficacy served as a predictor for teacher resilience (Ee and Chang, 2010; Raath et al., 2016; Ngui and Lay, 2020; Yada et al., 2021), However, to our knowledge, only a handful of studies have explored links specifically to their emotional resilience. One of these is a study by Daniilidou et al. (2020) in which the authors applied the Multidimensional Teacher Resilience Scale (Mansfield and Wosnitza, 2015) in a survey of 636 Greek primary school teachers and revealed that teacher self-efficacy predicted their emotional resilience. Significant positive relations between teacher self-efficacy and emotional resilience were found in yet another study conducted with pre-service teachers from Germany, Ireland, Malta, and Portugal (Peixoto et al., 2018).

In our study we followed the Job Demands-Resources approach (Bakker and Demerouti, 2017) and analyzed feedback, autonomy, social support, and opportunities for development as a set of workplace-related resources, and self-efficacy as a potential personal resource for teachers’ emotional resilience.

### 2.3 Emotional resilience and well-being

Research on teacher well-being is well established, yet it remains relevant and researcher attention to teacher well-being has not decreased. From a psychological point of view, well-being is not a stable, unchanging phenomenon. It is a generalized, positive internal state that can shift with changes in the person or external conditions (Yin et al., 2016). Due to fundamental changes in the teaching process and in working conditions, teacher well-being received considerable research attention during the COVID-19 pandemic and the post-pandemic period (Sacré et al., 2023). There was a need to understand and accept changes in teachers’ work and to adapt to the changed circumstances. This included examination of “disrupted” former rhythms, requirements to develop new skills (e.g., the use of information technologies; Sá et al., 2021) which impacted a teacher’s sense of themselves, their mental health, and more broadly–their well-being (Gutentag and Asterhan, 2022). A systematic review of studies on teacher well-being revealed that it can have a positive effect on teachers’ performance results, job satisfaction, teaching behavior, relationships with colleagues and students (Dreer, 2023), and can decrease teacher stress and burnout (Burić et al., 2019). Well-being can even be identified as one of the key indicators or criteria for assessing the effectiveness of changes in schools and in the work of teachers.

Work-related well-being is often referred to with various synonyms: workforce well-being, workplace well-being, occupational well-being, employee well-being. It can also be examined in the context of a specific professional domain, such as teacher well-being. Van Horn et al. (2004) were among the first to create a model of occupational well-being which refers to an individual’s “positive evaluation of various aspects of one’s job, including affective, motivational, behavioral, cognitive and psychosomatic dimensions” (p. 366). Viac and Fraser (2020) presented a basic definition of teacher well-being, that includes “teachers’ responses to the cognitive, emotional, health and social conditions pertaining to their work and their profession” (p. 18). Granziera et al. (2023) conceptualized well-being as “teachers’ evaluations of and functioning in their work environment” (p. 280). In summary, it can be said that teacher well-being is based on and reflects healthy functioning and effective work performance. From a wider perspective, workplace well-being is inextricably linked to employees’ psychological well-being and, more broadly, life well-being. In our study, we applied the approach to well-being developed by Zheng et al. (2015). The authors provided theoretical and empirical support for the employee well-being model, which combines workplace, psychological and life well-being types, and developed an empirically validated multidimensional well-being assessment instrument.

A considerable amount of research data has been accumulated on the multifaceted interrelationships between teacher resilience and well-being. Hascher et al. (2021) recently reviewed 46 publications from 2010 to 2020 and identified four strands of research describing the links between teacher resilience and well-being. The first strand includes publications that analyze resilience and well-being as similar constructs; the second analyses these constructs as a component of each other, with resilience subsumed into well-being or vice versa; the third strand analyses well-being as a predictor of teacher resilience; and the fourth highlights the role of resilience in the development of teacher well-being. For example, Burić et al. (2019) found that teacher resilience predicts lower levels of negative emotions, burnout and psychopathological symptoms and summarized that resilience acts as a protective factor for their well-being. On the other hand, it is important to underline that there is not enough research examining the links between teacher emotional resilience and well-being. Studies confirm that job resources help employees to maintain work engagement, motivation and stimulate teacher well-being (Skaalvik and Skaalvik, 2018; Benevene et al., 2020; Han et al., 2020; Granziera et al., 2023). These links can be more complex than the direct links between well-being and the work and personal aspects that strengthen it. One of the few available studies is one by Chen and Lee (2022), which was conducted with a sample of 407 teachers from Hong Kong, SAR and mainland China. It was found that teacher emotional resilience affected well-being directly and indirectly as a mediator in the relationships between school support and well-being.

### 2.4 Emotional resilience and intention to leave

Teachers tend to change schools or even leave the profession because of unappealing working conditions, insufficient funding, heavy workloads, lack of autonomy at work, and little support from management (Mansfield et al., 2016; Cordingley and Crisp, 2020; Howson, 2020; See et al., 2020; Worth and Van den Brande, 2020; Sabina et al., 2023). The first 5 years of teaching are particularly challenging for early-career teachers and, according to Gallant and Riley (2014), in many countries 40–50% of teachers leave the profession within that time. Therefore, research on individuals’ intention to leave and organizational strategies to increase teacher retention have become important tasks of the education system and of each educational institution.

The worldwide teacher shortage has encouraged researchers to examine what causes teachers to stay or leave (Kurtz and Maurice, 2018; Li and Yao, 2022; Tikkanen et al., 2022). In Lithuania, as in other countries, the COVID-19 pandemic and the turbulent geopolitical situation have led to an increase in the number of teachers leaving their jobs and a shortage of teachers in particular subject areas.

There are two subcategories of research in this field. Some focus on a teacher’s intention to leave the profession. We found this to be too broad an interpretation of intention to leave because the reasons for leaving the profession can be related to the person’s attitude to the profession and not to a specific school. Studies on teachers’ intentions to leave a school situation are more in line with current thinking that individual workplaces and organizations create specific conditions that enhance or restrain teacher resilience (Ungar et al., 2013; Wang et al., 2022). A teacher may find conditions in one school to be unacceptable, but moving to a new workplace may alleviate the reasons for leaving the profession. Recent research has also looked at a phenomenon called *teacher churn* when teachers change grade levels, subject areas, or schools (Dhaliwal et al., 2023) to find a better “fit.” While this may be an apt partitioning of intention to leave for a future study, we focus on the primary workplace with which our study participants identified themselves. Vekeman et al. (2017) compare the two types of intention to leave studies, focusing on person-organization (P-O) fit. Their analysis revealed that P-O fit is directly related to the intention to move to another school, but there was no direct relation between P-O fit and intention to leave the profession.

Studies show that intention to leave is negatively predicted by perceived organizational support and continuance commitment (Esop and Timms, 2019), which is based on costs related with leaving the organization (Hackett et al., 1994); work engagement (da Silva et al., 2021; Tvedt et al., 2021), job satisfaction (Räsänen et al., 2020). Other studies point to meaningful work, and valuation of teacher dignity, which diminish turnover intentions (Janik and Rothmann, 2015; Heleno et al., 2018).

Intention to leave studies are related to much more exhaustive research on teacher burnout, in which emotional exhaustion and the loss of emotional resources are exhibited (de Vera et al., 2019; Annamalai, 2022). Madigan and Kim (2021) conducted a meta-analysis of the effects of teacher burnout and job satisfaction on intentions to quit and concluded, that both phenomena are related with turnover intention, however, the negative effect of burnout on intention to leave is stronger compared to job satisfaction.

A broader meta-analysis of teachers’ intention to leave assumptions was provided by Li and Yao (2022), who examined 94 studies over the last 30 years. The authors found that teachers’ commitment, job satisfaction, work engagement, intrinsic motivation, and burnout were the strongest predictors of turnover intention. While all of the mentioned research foci are tangentially related to our research, we are looking for a more direct link between teacher emotional resilience and intention to leave. De Neve and Devos (2017) investigated how numerous factors, one of which was affective commitment, influence turnover intentions. Their path analysis revealed that teacher self-efficacy and affective commitment to a school directly reduced 272 Flemish teachers’ intention to leave the job. This is in line with the work of Meyer et al. (2002), who named three forms of organizational commitment, noting that affective commitment had the strongest negative correlation to intention to leave. Arnup and Bowles (2016) surveyed 160 Australian teachers with less than 10 years of experience and found that lower job satisfaction and a lower level of general resilience predicted intention to leave the teaching profession.

Our review of research reveals that teacher resilience, along with job and personal resources, can strengthen their well-being, relationships with school, and reduce turnover intentions. Unsatisfactory working conditions, high demands, limited opportunities to achieve professional goals, create a context that negatively affects the meaningfulness of teaching. If a person lacks emotional resilience, the internal capacity to adapt, manage or cope with emotionally demanding situations, this can be an obstacle to achieving goals, and can lead to feelings of dissatisfaction or mistrust. This confirms the need for research on teachers’ emotional resilience and the resources that strengthen it.

## 3 The current study

In this study we aimed to analyze the relationships between teachers’ emotional resilience, job and personal resources, well-being and intention to leave school. More specifically, resources were studied as antecedents to emotional resilience. Furthermore, emotional resilience was analyzed as a potential antecedent to teachers’ well-being and intention to leave, and also as an intervening variable (mediator) in the relationships between job and personal resources and two outcomes–well-being and intention to leave school.

*Research objectives*:

1. To examine job resources and self-efficacy as predictors of teacher emotional resilience.
2. To analyze job resources, teacher self-efficacy and emotional resilience as predictors of teacher well-being and intention to leave.
3. To investigate the mediating effect of teacher emotional resilience on the relationship between work-related resources and self-efficacy as independent variables and teacher well-being as a dependent variable.
4. To investigate the mediating effect of teacher emotional resilience on the relationship between work-related resources and self-efficacy as independent variables and intention to leave as a dependent variable.

## 4 Materials and methods

### 4.1 Data collection procedure and participants

Data were collected using convenience sampling and included 522 teachers working in Lithuanian primary and secondary schools located in cities, towns and villages of Lithuania. The main criteria for inclusion in the sample was a degree or certification in education and at least 1 year of experience in a teaching position. 91.6% of the sample were women, 5.4% were men, 3.1% did not indicate gender. The average age was 50.5 years (from 20 to 73 years, SD = 9.6), the average number of years of teaching experience was 26.4 years (SD = 10.8). 99.6% of the participants indicated that they have a higher education degree and 80.4% said that they work in schools located in cities.

An online self-administered questionnaire was used to collect data. The questionnaire was not publicly available–only teachers who received information about the study and an invitation to respond could participate. Information and the invitation were distributed by direct professional contacts via e-mails and social networks. We also asked school principals to disseminate information directly to school personnel. In the cover letter we presented the purpose of the study and provided instructions for completing the questionnaire. Participants were informed that the study was conducted in accordance with research ethics requirements, that participants’ responses were analyzed in aggregate for scientific purposes only, and that confidentiality of responses was guaranteed. We indicated that participation in the study is voluntary and that respondents could withdraw from the study at any time. Since none of the participants withdrew, the responses of all teachers in the sample were included in the final data set.

### 4.2 Research instruments

The questionnaire consisted of demographic questions on respondents’ age, gender, education, years of teaching experience, school location, and assessment scales for research variables.

*Emotional resilience* was measured using the Emotional Resilience scale from the Teacher Resilience Questionnaire, Version 1.5 (Mansfield and Wosnitza, 2015). The scale consists of four items, for example, “When I feel upset or angry at school, I can manage to stay calm.” Responses are scored on a five-point Likert scale, with 1 representing “Strongly disagree” and 5 – “Strongly agree.”

*Job resources* were measured using a composite indicator, consisting of four types of work-related resources (autonomy, feedback, social support, and opportunities for development) taken from the Job Demands–Resources Questionnaire (Bakker, 2014). Autonomy was measured using three items (“Can you participate in decision-making regarding your work?”). Feedback was assessed using three items (“My job offers me opportunities to find out how well I do my work.”). Social support was measured using three items (“If necessary, can you ask your colleagues for help?”) and opportunities for development were assessed using three items (“In my work, I have the opportunity to develop my strong points.”). Answers for autonomy, feedback and social support scales ranged from 1 point – “never” to 5 – “very often,” and for opportunities for development from 1 point – “strongly disagree” to 5 – “strongly agree.” We calculated the scores for every job resource scale, and the construct validity of the modeled composite job resources measure was evaluated by applying Principal component factoring with Varimax rotation when indicators of four job resource types were included as separate variables. One factor was obtained to explain 57.33% of data variance (KMO = 0,773; Bartlett’s Test of Sphericity Chi-Square = 452,477, *p* < 0,001).

*Self–efficacy* was assessed using the short version of the Occupational Self-Efficacy Scale (Schyns and Von Collani, 2002; Rigotti et al., 2008). A variety of self-efficacy scales related to specific activities and tasks have been used in research, and for this study we chose a scale related to the occupational domain. The scale consists of six items (“When I am confronted with a problem in my job, I can usually find several solutions.”). Responses were indicated on a five-point Likert scale ranging from 1 point – “strongly disagree” to 5 points – “strongly agree.”

*Well-being* was measured by applying the Employee Well-Being scale (Zheng et al., 2015) consisting of 18 items. The statements in the scale cover three areas of employee well-being–workplace well-being (“Work is a meaningful experience for me.”), psychological well-being (“I feel I have grown as a person.”), and life well-being (“I am close to my dream in most aspects of my life.”). Answers ranged from 1 point – “strongly disagree” to 5 points – “strongly agree.”

*Intention to leave* the school was measured using the Chiu and Francesco (2003) three-item scale (“In the last few months, I have seriously thought about looking for a new job.”). The respondents indicated their responses on a five-point Likert scale ranging from 1 point – “strongly disagree” to 5 points –“strongly agree.”

The questionnaire was administered in Lithuanian, translation of the items from English to Lithuanian was prepared by professional translators. The Lithuanian version of the Occupational Efficacy Scale was taken from a study presented by Žukauskaitė et al. (2019).

### 4.3 Statistical analyses

Data were analyzed using the IBM SPSS 27: descriptive statistics and reliability of the study measures were estimated; multiple regression models were tested to reveal the effect of job resources and self-efficacy in predicting teachers’ emotional resilience and to analyze predictors of two dependent variables–teacher well-being and intention to leave school. The PROCESS Macro tool – Model 4 (Hayes, 2013) was applied to test four mediation models with job resources and self-efficacy as independent variables, emotional resilience as a mediator, and both well-being, and intention to leave as dependent variables. 95% confidence intervals were estimated by using the bootstrapping technique with 5,000 bootstrap samples. The indirect effect through the mediating variable was confirmed if the effect’s 95% confidence interval did not include 0.

## 5 Results

Presentation of the research results corresponds to the stated objectives. Descriptive statistics of the study variables are presented in Table 1, followed by the results of three multiple regression models which tested: firstly, the influence of job resources and self-efficacy on teacher emotional resilience as a dependent variable; secondly, the role of both resources and emotional resilience in predicting teacher well-being and intention to leave as dependent variables (Table 2). Finally, aligned with the third and fourth study objectives, the results of four mediation models are presented that highlight the mediating role of emotional resilience in the links between work-related and personal resources (independent variables) and teacher well-being and intention to leave (Table 3) as dependent variables.

**Table 1:** Means, standard deviations, intercorrelations between variables and scales’ reliability indicators (*n* = 522).

	Variables	1	2	3	4	5
1	Emotional resilience	(0.733)				
2	Job resources	0.449^**^	(0.747)			
3	Self-efficacy	0.503^**^	0.517^**^	(0.861)		
4	Well-being	0.490^**^	0.555^**^	0.661^**^	(0.913)	
5	Intention to leave	−0.231^**^	−0.452^**^	−0.247^**^	−0.356^**^	(0.882)
*M*		3,429	3,976	3,873	3,980	2,094
*SD*		0.654	0.562	0.499	0.476	0.968

**p < 0.001. M, mean; SD, standard deviation; Cronbach’s alpha coefficients are presented in the diagonal.

**Table 2:** Multiple regression models testing predictors of teacher emotional resilience, well-being and intention to leave.

Independent variables	Dependent variables											
	Emotional resilience Model 1				Well-being Model 2				Intention to leave Model 3			
	β	*t*	*p*	VIF	β	*t*	*p*	VIF	β	*t*	*p*	VIF
Job resources	0.258	6.023	0.000	1.365	0.252	6.815	0.000	1.561	−0.434	−9.171	0.000	1.461
Self-efficacy	0.370	8.625	0.000	1.365	0.457	11.946	0.000	1.461	−0.005	−0.112	0.911	1.561
Emotional resilience					0.147	4.016	0.000	1.432	−0.033	−0.714	0.476	1.432
	*R^2^* = 0.302 *Adj R^2^ =* 0.299; *F* (2,521) *=* 112.232, *p =* 0.000				*R^2^* = 0.514 *Adj R^2^ = *0.512; *F* (3,521) *=* 182.889, *p = *0.000				*R^2^* = 0.205 *Adj R^2^ = *0.201; *F* (3,521) *=* 44.613, *p = *0.000			

TR, teacher resilience; VIF coefficients for all independent variables in every model did not exceed statistical level of 2.0.

**Table 3:** Mediation analysis results for dependent variables – well-being and intention to leave.

Part 1. Job resources, emotional resilience and well-being						Part 2. Job resources, emotional resilience and intention to leave					
Independent variables/Effects	*b*	*SE*	*p*	*95% CI*		Independent variables/Effects	*b*	*SE*	*p*	*95% CI*	
				*LLCI*	*ULCI*					*LLCI*	*ULCI*
JR → WB	0.354	0.033	< 0.001	0.290	0.418	JR → ITL	−0.751	0.075	< 0.001	−0.899	−0.603
JR → ER	0.523	0.046	< 0.001	0.433	0.612	JR → ER	0.523	0.046	< 0.001	0.433	0.612
*R^2^* = 0.202, *F*(1,520) = 131.508, *p* < 0.001						*R^2^* = 0.202, *F*(1,520) = 131.508, *p* < 0.001					
JR → ER → WB	0.220	0.028	< 0.001	0.164	0.275	JR → ER → ITL	−0.052	0.065	0.420	−0.180	0.075
*R^2^* = 0.381, *F*(2,519) = 159.442, *p* < 0.001						*R^2^* = 0.205, *F*(2,519) = 67.041, *p* < 0.001					
Total effect	0.469	0.031	< 0.001	0.408	0.529	Total effect	−0.778	0.067	< 0.001	−0.910	−0.646
*R^2^* = 0.308, *F*(1,520) = 231.117, *p* < 0.001						*R^2^* = 0.204, *F*(1,520) = 133.520, *p* < 0.001					
Direct effect	0.354	0.033	< 0.001	0.290	0.418	Direct effect	−0.751	0.075	< 0.001	−0.899	−0.603
Indirect*effect	0.115	0.018		0.081	0.153	Indirect*effect	−0.027	0.036		−0.101	0.044
Part 3. Self-efficacy, emotional resilience and well-being						Part 4. Self-efficacy, emotional resilience and intention to leave					
SE → WB	0.529	0.035	< 0.001	0.460	0.598	SE → ITL	−0.339	0.095	< 0.001	−0.525	−0.153
SE → ER	0.659	0.049	< 0.001	0.562	0.757	SE → ER	0.659	0.049	< 0.001	0.562	0.757
*R^2^* = 0.253, *F*(1,520) = 176.231, *p* < 0.001						*R^2^* = 0.253, *F*(1,520) = 176.231, *p* < 0.001					
SE → ER → WB	0.153	0.027	< 0.001	0.101	0.206	SE → ER → ITL	−0.212	0.072	0.004	−0.354	−0.070
*R^2^* = 0.471, *F*(2,519) = 230.889, *p* < 0.001						*R^2^* = 0.076, *F*(2,519) = 21.435, *p* < 0.001					
Total effect	0.630	0.031	< 0.001	0.568	0.691	Total effect	−0.479	0.082	< 0.001	−0.640	−0.317
*R^2^* = 0.438, *F*(1,520) = 404.560, *p* < 0.001						*R^2^* = 0.061, *F*(1,520) = 33.751, *p* < 0.001					
Direct effect	0.529	0.035	< 0.001	0.460	0.598	Direct effect	−0.339	0.095	< 0.001	−0.525	−0.153
Indirect*effect	0.101	0.020		0.065	0.143	Indirect*effect	−0.140	0.050		−0.250	−0.048

*Based on 5,000 bootstrap samples; JR, job resources; WB, Well-being; ER, emotional resilience; ITL, intention to leave; SE, self-efficacy; b, unstandardized regression coefficients; SE, standard errors; CI, confidence interval for b.

The results revealed that the intercorrelations between the study variables are statistically significant: positive correlations were received for the relationships among emotional resilience, self-efficacy and well-being, and negative correlations for the relationships of all three variables with intention to leave. Emotional resilience most strongly correlated with self-efficacy and well-being (*r* = 0.503, *p* < 0.001 and *r* = 0.490, *p* < 0.001, respectively). Job resources had the strongest negative correlation with intention to leave (*r* = −0.452, *p* < 0.001).

The associations of emotional resilience, work resources, self-efficacy, well-being, and intention to leave with the demographic characteristics of the sample were tested using correlation analysis for age and teaching experience; the Student’s t-test was used for the comparison of means between gender groups, and the ANOVA test for groups of respondents divided according to education level and school location. Age and years of teaching experience were found to correlate only with job resources (*r* = 0.095, *p* < 0.05 for age; *r* = 0.130, *p* < 0.01 for teaching experience) and intention to leave (*r* = −0.115, *p* < 0.05 for age; *r* = −0.100, *p* < 0.05 for teaching experience). The means of groups divided by gender, education and school location fluctuate around the mean value obtained for the whole sample, the differences are marginal and statistically non-significant. In view of these results, demographics were not included in further analysis of the data.

To examine job resources and self-efficacy as predictors of teacher emotional resilience we tested a multiple regression model, in which job resources and self-efficacy were included as independent variables and emotional resilience as a dependent variable. The results are presented in Table 2. In the other two regression models well-being and intention to leave were included as dependent variables, and job resources, self-efficacy, emotional resilience as independent factors. Results are presented in Table 2.

Thus, job resources and a personal resource–self-efficacy positively predicted teacher emotional resilience. As shown in Model 1, two predictors explained 30.2% of the variance of the dependent variable, standardized beta coefficients were positive and significant both for job resources and self-efficacy (*β* = 0.258, *p* < 0.001 and *β* = 0.370, *p* < 0.001, respectively). Emotional resilience as a third independent variable was added in Models 2 and 3 to test predictors of well-being and intention to leave. The results revealed that Model 2 explains 51.4% of the well-being variance, all three predictors were significant, however, their effects were different. Self-efficacy was the strongest predictor of well-being, followed by job resources and emotional resilience (*β* = 0.457, *p* < 0.001, *β* = 0.252, *p* < 0.001 and *β* = 0.147, *p* < 0.001, respectively). The independent variables explained 20.5% of the intention to leave variance (Model 3), however, only job resources were a significant predictor, while self-efficacy and emotional resilience showed nonsignificant results.

Through the third and fourth objectives of the study, we sought to determine whether emotional resilience acts as an intermediate variable (mediator) in the links of work-related resources and self-efficacy with teacher well-being and intention to leave. Four mediation models were calculated using the Hayes (2013) Process Macro tool v. 4.0, Model 4. The independent variables are job resources and self-efficacy, the dependent variables are well-being and intention to leave, and emotional resilience is introduced as a mediating variable. The results are presented in Table 3.

In this part of the analysis, we explored the role of emotional resilience as a mediating variable for job resources and self-efficacy in predicting teacher well-being and intention to leave the school. The results show the significant indirect effect of emotional resilience on the positive relationships between job resources and well-being (Table 3, Part 1) and for self-efficacy and well-being (Table 3, Part 3). Job resources together with emotional resilience predicted 30.8% of well-being variation. Both direct and indirect effects of job resources were significant (*b* = 0.354, *p* < 0.001 and *b* = 0.115, *CI* [0.081; 0.153], respectively). We obtained similar results when the independent variable was self-efficacy. The total effect of the positive impact of self-efficacy together with emotional resilience on well-being was high (*b* = 0.630, *p* < 0.001) and both variables explained up to 43.8% of well-being variation. As in the case when the independent variable was job resources, the direct and indirect effects of self-efficacy on well-being were significant (*b* = 0.529, *p* < 0.001 and *b* = 0.101 *CI* [0.065; 0.143], respectively). To conclude, teacher emotional resilience mediated the positive relationships between job resources and teacher well-being and also between self-efficacy and well-being. Job resources and self-efficacy strengthen teacher well-being not only directly, but also via emotional resilience as a mediating variable.

The results of the indirect effect of emotional resilience on the relations between job resources and intention to leave (Table 3, Part 2) revealed that job resources negatively impact the intention to leave the school directly, whereas the mediating effect of emotional resilience in this relationship was insignificant (*b* = −0.751, *p* < 0.001 and *b* = −0.027, *CI* [−0.101; 0.044], respectively). Meanwhile the mediating effect of emotional resilience for the relations between self-efficacy and intention to leave were confirmed: the negative direct impact of self-efficacy on the intention to leave school is complemented by the indirect negative effect of emotional resilience (*b* = −0.339, *p* < 0.001 and *b* = −0.140, *CI* [−0.250; −0.048], respectively). Self-efficacy reduces the level of teachers’ intention to leave directly and via emotional resilience as the mediating factor in this relationship.

## 6 Discussion

Job resources and self-efficacy were shown to be significant predictors for teachers’ emotional resilience, as teachers who assigned higher values to these factors had higher emotional resilience scores. This means that job resources – which in our study combine autonomy, performance feedback, social support and opportunities for development – along with occupational self-efficacy can be identified as emotional resilience resources. Such confirmation, in our opinion, is important both empirically and practically. Seeing the regulatory role of labor resources and personal self-efficacy, we have confirmed their dynamic capacity to nurture emotional resilience. The latter, in its essence and content, is the central personal force that guarantees a teacher’s ability to withstand challenges, to guard against burnout (Tait, 2008; Evans-Palmer, 2010; Mansfield et al., 2012; Gu and Day, 2013). This is important in everyday practice as demanding context prevails in teachers’ work. Beutel et al. (2019) called teaching a “take home” job, since the intense workload is felt by teachers not only during the working day, but also during their free time.

The added value of our research was to confirm prognostic links of both external and internal resources to emotional resilience. This filled a gap in the research field. The concurrent connections between teachers’ self-efficacy, resilience, and an active personal stance toward the teachers’ job were underexplored. To date, most of the accumulated evidence has been on the isolated impact of inadequate job resources alone on teacher burnout (Hakanen et al., 2006; Manuti et al., 2022), when other soft power resources, such as self-efficacy were excluded. Similarly, when examining the relationship of the latter construct with resilience decoupled from job resources, it was found that self-efficacy served as a predictor for teacher resilience (Raath et al., 2016; Ngui and Lay, 2020; Yada et al., 2021). Self-efficacy and resilience were found to be collaterally important for teachers’ behavior (Wilcox and Lawson, 2017) or for teacher burnout (Fathi and Saeedian, 2020). Studies in the last decade have highlighted other possible relationships, for example it was found that resilience was a predictor of early teachers’ self-efficacy (Johnson et al., 2014; Gratacos et al., 2021). Most studies on self-efficacy as a predictor of resilience focused on a sample different from ours, e.g., Lightsey (2006) looked at the youth population. Finally, educational research has not answered questions about in-service teachers’ self-efficacy as a prerequisite for resilience, although a number of studies have confirmed these links. Ee and Chang (2010) found that self-efficacy is an antecedent of resilience in a study of pre-service teachers. Ngui and Lay (2020) also studied pre-service teachers. We have complemented the research on the relationship between job and personal resources and individual and organizational outcomes by revealing how they operate in specific teaching contexts, a point made by Sokal et al. (2020).

We examined the implications of teachers’ emotional resilience for their well-being from two perspectives: firstly, we evaluated the potential of emotional resilience for the prediction of well-being along with job resources and self-efficacy, and secondly, we examined the importance of emotional resilience as an intermediate variable (mediator) in the links of job and personal resources and well-being. Emotional resilience was found to be a direct positive predictor of teacher well-being; however, its impact is lower (*β* = 0.147, *p* < 0.001) compared to job resources and self-efficacy (*β* = 0.457, *p* < 0.001 and *β* = 0.252*, p* < 0.001, respectively). Testing the mediating effect of teacher emotional resilience on the relationships between work-related resources and self-efficacy as independent variables and teacher wellbeing as a dependent variable revealed that emotional resilience acts as a partial mediator in these relationships: job resources and self-efficacy reinforces teachers’ well-being directly and via emotional resilience as well. The indirect effect of independent variables on well-being through emotional resilience is significant both for job resources (*b* = 0.115, [0.081, 0.153]) and self-efficacy (*b* = 0.101, [0.065, 0.143]; see Table 3, Part 1 and Part 3). An analysis of research literature revealed that research on the links between the phenomenon of general teacher resilience and teacher well-being is sufficiently established (Gibbs and Miller, 2014; Mansfield et al., 2016; Gray et al., 2017; Burić et al., 2019). However, we emphasize that emotional resilience has not yet received enough attention from researchers. The implications of emotional resilience for teacher well-being are just beginning to be explored. One of the few such studies is that of Chen and Lee (2022), who revealed that teachers’ emotional resilience predicts well-being not only directly, but also acts as a mediator in the relationships between school support and teacher well-being. The links between teachers’ emotional resilience and well-being revealed in our study support these results.

Unlike teacher well-being, teachers’ intentions to leave school are not desirable organizational outcomes. This intention can lead to actual behavioral decisions and the termination of working relationships with the school. However, even before quitting, the intention to leave can negatively affect work motivation, work engagement, colleagues, and the overall psychological climate. Our study shows that job resources, self-efficacy and emotional resilience have significant negative direct relations with teachers’ intention to leave (see Table 1). However, multiple regression analysis with all three variables as predictors for the intention to leave showed that job resources alone were significant in predicting this intention (*β* = −0.434*, p* < 0.001; see Table 2, Model 3). Teachers who value the school’s job resources more highly (possibility to receive results-based performance feedback, work autonomy, social support, and opportunities for professional development) are less likely to leave school and look for another job. The aforementioned research variables are directly negatively correlated with intention to leave, however the effects of self-efficacy and emotional resilience in predicting intention to leave were insignificant. Only perceived job resources directly and negatively predicted teachers’ turnover intentions. The mediating effect of emotional resilience on the relationship between work and personal resources and intention to leave was also not confirmed (see Table 3). Thus, emotional resilience is negatively associated with intention to leave but does not predict it and does not have a mediating effect in the links between job and personal resources with intention to leave. This study found that teacher turnover depends more on external factors of the school and work environment than on their individual characteristics. The importance of the working environment and conditions for teachers’ intention to leave is also revealed in meta-analytical reviews (Li and Yao, 2022). Some studies suggest that not only direct but also more complex connections may exist between teachers’ emotional resilience and the intention to leave. For example, Lee et al. (2021) in a sample of secondary physical educators found that teacher resilience was related with turnover intention through a negative association with the mediator of emotional exhaustion.

The results of our study are aligned with Theory of Planned Behavior, which states that attitudinal orientations have the greatest influence on a person’s behavioral intentions: various external and internal factors first prompt certain dispositions, which then impact the behavioral intentions. We studied direct relations between workplace and personal variables with the intention to leave school without taking into account teachers’ attitudes toward the school at which they are employed. According to the Theory of Planned Behavior, affective aspects are one of the factors that shape behavioral attitudes and intentions (Ajzen, 2020; Bosnjak et al., 2020), therefore, in future studies it would be appropriate to analyze in more detail the significance of emotional resilience in teachers’ turnover intentions, taking into account their attitudes toward the school.

## 7 Limitations and guidelines for future studies

Evidence for our results came from a rather homogenous sample of Lithuanian teachers, predominantly urban women with considerable work experience. Therefore, the narrow distribution limits the external validity of our findings. Further research is needed to explore to what extent our findings are applicable for teachers with more diverse characteristics. The other shortcoming of our study is related to a single source of information, namely teachers’ self-reporting. The content of items addressed *ex post facto* opinion regarding one’s own ability to maintain a positive stance, to make use of personal resources and agency. Reports on a teacher’s daily experience might provide a more explicit picture of emotional resilience-in-act. Moreover, all these constructs are not stable and fixed. Periodic or follow-up assessments at other times could maximize the ecological validity of our findings and predict behavior of teachers in real-life settings.

Future research should focus on a wider range of personal and work environment variables and their interaction with teacher resilience. For instance, professional commitment or the understudied phenomenon of professional calling deserve a more detailed investigation. Meaning-making findings among working teachers could supplement this field of research. Beyond surveys, qualitative data documenting teachers as creators of meanings would increase understanding about forces that sustain inner equilibrium and commitment to a professional pathway. Recent findings of teaching during lockdown (Narayanan and Ordynans, 2022) once more reminded us that a proper study of man is incomplete without self-reflection about the purpose of one’s own activities in current life situation. Beyond surveys, qualitative data documenting teachers as creators of meanings would increase understanding about forces that sustain inner equilibrium and commitment to a professional pathway.

We focused on teachers’ perception of their work setting. However, it might be relevant to analyze the work environment, school type as well as the culture and climate of a specific school, since generalized, non-evidence-based recommendations do not always correspond to the real situation at particular schools.

Many studies examine teacher burnout and retention for specific subject areas. Special education appeared to be an area that is particularly challenging (Kerr and Brown, 2015; Bettini et al., 2020). There were variabilities when comparing work experience with beginning teachers intending to leave in greater numbers than their experienced counterparts (De Neve and Devos, 2017; Chambers et al., 2019). In our opinion, this particular vector of research should be extended. More complex studies are also needed, covering assumptions regarding teachers’ long-term relations not only with the school but also with the profession in association with individual, organizational and family relations.

## 8 Implications

Who should be held responsible for developing emotional resilience and providing the resources that will strengthen teachers’ capacities to cope? Clearly, there is no single solution, but a variety of interventions throughout a teacher’s tenure are necessary. Developing individual emotional resilience is important, but we also need to consider the organizational and system-wide conditions in which a teacher works to consider whether those conditions hinder or promote emotional resilience.

Firstly, we might consider who should enter the teaching profession. According to Aguilar (2018a), before considering how to cultivate emotional intelligence we need to identify emotional intelligence and resilience in prospective teachers. She states that building emotional intelligence and resilience can take longer and be more complex than building pedagogical knowledge and skill. This is in line with claims by earlier researchers that prospective teachers cannot develop attitudes and dispositions within the time frame of a teacher education program unless they bring certain dispositions with them into the programs (Jacobowitz, 1994; Denner et al., 2001). Chambers et al. (2019) also assert that some of the factors associated with intention to leave are not easily modifiable, but certain interventions can reduce teacher attrition.

Increasingly, non-academic attributes have been named essential for success and professionalism in teaching. Once teachers have entered a teacher education program, it is crucial that pre-service teachers receive instruction, mentoring and coaching on how to recognize, appreciate, and respond to emotions (Aguilar, 2018a, b). Studies conducted around the world confirm that teacher education can play a crucial role in the resilience developing process (Hammond, 2004; Day and Gu, 2014).

Once teachers have entered the workforce, leaders of organizations and educational systems need to be mindful of their responsibility to provide both external and internal resources for emotional resilience, not leaving teachers to cope on their own (Hamid and Ghazali, 2018). Providing job resources and eliminating sources of stress is not enough. Leaders also need to provide professional development programs that will strengthen various personal characteristics. Our study revealed several personal and organizational resources of emotional resilience: occupational self-efficacy, possibilities to receive feedback, autonomy at work, social support, opportunities for professional development. The Conservation of Resources Theory argues that resilience, like other stress coping resources, is not only depleted but also needs to be replenished and sustained (Hobfoll, 1989; Hobfoll et al., 2018).

Clearly, there are many players that should provide professional development, support, and resources for building emotional resilience. The process begins in teacher education programs and continues in schools that create a workplace environment conducive to optimal teacher performance and low turnover. On the other hand, teachers are not just passive consumers of resources provided by the school or educational system. Teachers’ self-leadership, mindfulness, active capacity building for emotional resilience through experiential and professional skill-building programs, and proactivity in helping the school to focus its resources can also contribute to increasing the “basket” of emotional resilience resources.

## 9 Conclusion

The focus of this study was the emotional resilience of teachers – the internal capacity to adapt, manage or cope with emotionally demanding situations. We examined resilience resources and its implications for teacher well-being and intention to leave school. Our investigation revealed that perceived workplace characteristics – performance feedback, autonomy, social support, and opportunities for professional growth – along with self-efficacy were positively related with and predicted teacher emotional resilience. These can be listed as job and personal resources, which strengthen teachers’ capability to maintain emotional balance and effectively manage emotional reactions in challenging circumstances.

This study reveals, that teachers’ emotional resilience, job resources and teacher self-efficacy have a direct positive impact on teacher well-being. It also found that teacher emotional resilience mediated the positive relationships between job resources and teacher well-being and also between self-efficacy and well-being. Job resources and self-efficacy strengthen well-being directly and via emotional resilience as a mediating variable.

Emotional resilience, job resources and self-efficacy negatively correlated with teachers’ intention to leave school. However, only perceived job resources and self-efficacy were significant predictors of the intention to leave. Teachers who value the resources provided by the school, who have confidence in themselves and believe in their abilities to effectively perform in the profession. Are less likely to leave school. The role of emotional resilience as a mediator in the relationships between job and personal resources and intention to leave has not been established.

In summary, our study has revealed some of the resources of teachers’ emotional resilience and its links to teacher well-being and intention to leave, opening up possibilities for improving teachers’ working lives.

## Data availability statement

The datasets presented in this article are not readily available because the data set includes identifying data and cannot be released. Requests to access the datasets should be directed to dalia.bagdziuniene@fsf.vu.lt.

## Ethics statement

Ethical review and approval was not required for the study on human participants in accordance with the local legislation and institutional requirements. The studies were conducted in accordance with the local legislation and institutional requirements. The participants provided their written informed consent to participate in this study.

## Author contributions

DB: Conceptualization, Data curation, Formal analysis, Investigation, Methodology, Project administration, Resources, Software, Supervision, Validation, Visualization, Writing – original draft, Writing – review & editing. AK: Conceptualization, Investigation, Project administration, Resources, Supervision, Validation, Visualization, Writing – original draft, Writing – review & editing. DN: Conceptualization, Formal analysis, Resources, Supervision, Writing – original draft, Writing – review & editing. ES: Conceptualization, Formal analysis, Investigation, Resources, Supervision, Writing – original draft, Writing – review & editing.
